# Annotation-free prediction of immunotherapy response in melanoma using single-cell transcriptomic data

**DOI:** 10.1371/journal.pone.0343633

**Published:** 2026-02-27

**Authors:** Da Eun Oh, Gaeun Kee, Ji-Hye Oh, Wonkyung Kim, Young Gwang Kang, Chae Won Park, Tae Joon Jun, Chang Ohk Sung

**Affiliations:** 1 Bioinformatics Core Laboratory, Convergence Medicine Research Center, Asan Institute for Life Sciences, Asan Medical Center, Seoul, Republic of Korea; 2 Department of Internal Medicine, Asan Medical Center, University of Ulsan College of Medicine, Seoul, Republic of Korea; 3 Department of Medical Science, Asan Medical Center, University of Ulsan College of Medicine, Seoul, Republic of Korea; 4 Department of Information Medicine, Asan Medical Center, Seoul, Republic of Korea; 5 Department of Pathology, Asan Medical Center, University of Ulsan College of Medicine, Seoul, Republic of Korea; 6 Department of Medical Informatics and Statistics, Asan Medical Center, University of Ulsan College of Medicine, Seoul, Republic of Korea; Sichuan University, CHINA

## Abstract

Immune checkpoint inhibitors (ICIs) have transformed the advanced melanoma treatment landscape; however, a subset of patients achieve durable responses. Current biomarkers, such as PD-L1 expression and tumor mutational burden, offer limited predictive power due to the profound heterogeneity of melanoma. Accordingly, we developed an artificial intelligence (AI)-based model to predict ICI responsiveness using single-cell RNA sequencing (scRNA-seq) data without requiring cell type annotation. scRNA-seq data profiled using Smart-seq2 platform were downloaded from a public repository (GEO: GSE120575). From these data, we analyzed 16,290 tumor-infiltrating cells from melanoma scRNA-seq dataset. Various AI-based models, including Extreme Gradient Boosting, Random Forest, Logistic Regression, Support Vector Machine, Feedforward Neural Network, and Convolutional Neural Network were constructed, with the best-performing model achieving an area under the curve of 0.87. This AI-driven approach identified 29 key predictive biomarkers, including *CCR7* and *MTRNR2L2*. Validation using three independent bulk RNA-seq datasets (cBioPortal: DFCI melanoma; ENA: PRJEB23709; GEO: GSE91061) suggested that *CCR7* was associated with favorable ICI response and improved survival, whereas *MTRNR2L2* showed a tendency toward enrichment in non-responders and poorer outcomes. Cell-type-specific expression analysis revealed that *CCR7* was primarily expressed in B cells and memory T cells from responders, whereas *MTRNR2L2* was elevated in exhausted and cytotoxic T cells in non-responders. CCR7-positive B cells exhibited activation of the NF-κB pathway and demonstrated prognostic significance independent of the melanoma primary site or histologic subtype. However, among the three molecular subtypes, including immune, keratin, and microphthalmia-associated transcription factor (MITF)-low, *CCR7* expression was significantly associated with the immune subtype. Additionally, pathway-level deep learning models reinforced these findings, highlighting immune activation in responders and cell cycle-related signals in non-responders. Our study demonstrates that predictive modeling based on unannotated scRNA-seq data enables clinically relevant biomarker identification, offering a robust approach for patients with stratifying melanoma and guiding personalized immunotherapy.

## Introduction

Over the past decade, immunotherapy and targeted therapies have revolutionized the treatment landscape for advanced melanoma [1]. Immune checkpoint inhibitors (ICIs)—notably cytotoxic T-lymphocyte-associated protein 4 (CTLA-4) and programmed cell death protein 1 (PD-1) or programmed death-ligand 1 (PD-L1) inhibitors—alongside *BRAF* and mitogen-activated protein kinase (MEK) inhibitors, have been pivotal in this evolution [2,3]. These advances have led to unprecedented improvements in patient outcomes. Combining CTLA-4 and PD-1 inhibitors can achieve a median survival exceeding 60 months, representing a substantial breakthrough in oncology [4,5]. Despite these promising results, the benefits of immunotherapy are not universal, with a substantial proportion of patients exhibiting low or limited response rates [4,6]. Response variability is largely attributed to the complex and heterogeneous tumor microenvironment (TME) [7,8]. TME consists of malignant cells, diverse stromal cells, immune populations, and endothelial components that collectively play a crucial role in tumor progression, metastasis, and modulate therapeutic outcomes [3]. Among various immune cell types, CD8 ⁺ T cells contribute to maintaining immune equilibrium and are associated with improved survival outcomes following immunotherapy [9–11]. However, the cellular composition of TME is not yet fully understood, and new subtypes continue to be reported [12]. Therefore, analyzing all single cells within tumor tissues without prior cell type annotation may uncover previously unrecognized findings.

Given this variability in ICI treatment outcomes, identifying reliable biomarkers to predict which patients are most likely to respond well to immunotherapy is critical, enabling optimal candidate selection for personalized treatment [1,6]. Although PD-L1 expression and tumor mutational burden are well-established biomarkers for predicting responses to ICIs [13], their predictive accuracy is limited by the substantial intertumoral and intratumoral heterogeneity of melanoma, which influences the immune microenvironments and therapeutic outcomes [14]. This highlights the need for novel biomarkers that effectively capture the heterogeneity of melanomas and improve ICI response prediction.

This study aims to overcome these limitations by using single-cell RNA sequencing (scRNA-seq) to develop an artificial intelligence (AI)-based model for predicting immunotherapy responsiveness in patients with melanoma [15]. The scRNA-seq technology enables high-resolution analysis of gene expression at the individual cell level, allowing for the characterization of tumor-infiltrating lymphocytes and other immune populations within TME [16–18]. Therefore, AI models for predicting immunotherapy responses using scRNA-seq data have been attempted. However, utilizing the complex information of high-dimensional data from single cells remains complicated. Two common approaches are to simplify the data or to rely on single-cell annotation [19,20]. Although these strategies have clear advantages, loss of information may occur. In contrast, building predictive models without cell-type annotations enables implementation without reliance on predefined annotations. This approach could increase research flexibility, enable refined TME analysis, and facilitate the discovery of previously unrecognized immune cell response patterns.

Accordingly, we compared a range of machine learning and deep learning approaches. Tree-based ensemble models such as XGBoost and Random Forest were included because they can effectively capture nonlinear interactions in structured transcriptomic data and often yield strong predictive accuracy, while providing feature importance measures for interpretability [21]. Classical algorithms, such as logistic regression and support vector machines, were tested as baselines due to their simplicity, computational efficiency, and frequent use in biomedical prediction tasks, although they are less capable of modeling complex nonlinear relationships [22]. Neural network architectures, including feed-forward and convolutional networks, were incorporated to explore their potential in capturing complex, high-dimensional gene expression patterns, despite their high data and computational requirements and increased risk of overfitting [23]. By evaluating these complementary approaches, we aimed to balance interpretability and predictive performance in identifying robust biomarkers for immunotherapy response.

In this study, we demonstrate that immunotherapy response can be predicted based on scRNA-seq without cell type annotation. Using this model, potential biomarkers will be identified to select optimal patients most likely to respond to ICIs and improve melanoma heterogeneity characterization [15].

## Materials and methods

### scRNA-seq data used in AI prediction models

scRNA-seq and cell-type annotation data (accession number GEO: GSE120575) of melanoma samples from Sade-Feldman et al. [24] were obtained and analyzed. The scRNA-seq dataset includes 48 tumor samples from 32 patients with metastatic melanoma treated with ICIs. This dataset was generated by sorting CD45 ⁺ single cells and profiling gene expression using the Smart-seq2 protocol. The dataset had already been preprocessed and filtered, and we analyzed these filtered data without applying any additional ad hoc filtering. Briefly, as described by Sade-Feldman et al. [24], FASTQ files were aligned to the human reference genome (GRCh37/hg19) using STAR. Gene expression was quantified in transcripts per million (TPM) using RSEM. Quality control excluded (i) cells with zero expression of both CD45 and CD3E, (ii) cells expressing fewer than 1,000 genes, and (iii) cells whose mean log₂(TPM + 1) expression of housekeeping genes was < 2.5.

Our study was conducted using publicly accessible data that are openly available to anyone and do not contain any personally identifiable information. As such, our study was exempt from IRB review.

### scRNA-seq data visualization

The data (GSE120575) from a previous scRNA-seq study by Sade-Feldman et al. [24] of melanoma ICI immunotherapy were analyzed using the Seurat R package (version 4.4.0). We downloaded log2(TPM + 1) transformed expression values and used them for downstream analyses. As part of our quality control on the downloaded dataset, we confirmed that mitochondrial percentage was < 5% across cells. ICI treatment response data were available for each patient. The scRNA-seq data for each response group were as follows: responders (17 samples, 5,564 cells) and non-responders (31 samples, 10,726 cells). To identify high-variance genes, we selected genes with a variance > 6, following the same criterion used in a previous study [24], resulting in a total of 4,374 genes. These genes were used for clustering analysis for visualization. Briefly, principal component analysis (PCA) was used for dimensionality reduction, and the number of significant principal components was calculated using built ‘RunPCA’ function. The cells underwent unsupervised clustering, according to the shared nearest neighbor graph (‘FindNeighbors’ function, using the top 10 principal components (PCs); ‘FindClusters’ function, resolution = 0.5) and were visualized by uniform manifold approximation and projection (UMAP) using the top 10 PCs. We initially computed the first 10 principal components and used them in subsequent analyses. Cell type annotation information was obtained from the Sade-Feldman scRNA-seq study (GSE120575) [24].

### Differentially expressed gene profiling and pathway analysis in scRNA-seq dataset

Differentially expressed genes (DEGs) from all single cells (n = 16,290) were identified using the Wilcoxon rank-sum test, with an adjusted P value<0.05 according to the Benjamini–Hochberg (BH) method and a log2-fold change > 1. Specifically, DEGs were identified from the downloaded log2-transformed TPM values using the *Seurat* R package, using the FindAllMarkers and FindMarkers functions to derive DEGs for each group according to a previous study [25]. To identify the meaningful biological pathways related to DEGs, clusterProfilter (v4.6.2) R package was used for Reactome enrichment analyses. clusterProfiler was also used for Signatures, with a false discovery rate (FDR) or BH-adjusted *P*-value of <0.05 (hypergeometric test) were considered significantly enriched. The immune exhaustion score was calculated using the AddModuleScore function in the Seurat, based on a gene set associated with T cell exhaustion, including *PDCD1*, *CTLA4*, *LAG3*, *HAVCR2*, and *TIGIT* [26]. DEGs between CCR7-positive and CCR7-negative B cells were identified using the Seurat FindMarkers function with parameters min.pct = 0.1 and logfc.threshold = 0.25. Multiple testing correction was performed on the raw P values using the FDR method.

### Predictive model based on single-gene expression from scRNA-seq data

Six classification models were constructed to predict responders and non-responders based on gene expression data as follows: Extreme Gradient Boosting (XGBoost), Random Forest, Logistic Regression, Support Vector Machine (SVM), Feedforward Neural Network (FNN), and 1D Convolutional Neural Network (1D CNN). All six models use the same input format (cell by DEG expression matrix) of scRNA-seq data (S1A Fig). Feature scaling was performed using “StandardScaler.” The dataset was split into 80% training and 20% test sets, preserving the class distribution. For XGBoost, Random Forest, SVM, and FNN, hyperparameters were optimized using RandomizedSearchCV with stratified 5-fold cross-validation within the training set. The best models were selected based on receiver operating curve-area under the curve (ROC-AUC) score. The optimal models were then retrained on the full training set and evaluated on the test set. In contrast, the 1D-CNN architecture consisted of two Conv1D layers with ReLU activation, batch normalization, max pooling, and dropout regularization to reduce overfitting. The model was trained using the Adam optimizer with binary cross-entropy loss for up to 100 epochs, with early stopping (patient = 5). Model performance was evaluated using ROC-AUC for all classifiers, which was calculated on the held-out 20% test set, whereas accuracy and loss were specifically assessed for the 1D-CNN model. Feature importance was extracted from tree-based models (XGBoost and Random Forest). All analyses were performed in Python (version 3.10.12) using scikit-learn (version 1.6.1) for classical machine learning models and TensorFlow (v2.8.0) with Keras (v2.8.0) for deep learning models. For reproducibility, all random seeds were fixed to 42.

### Predictive model based on pathway level from scRNA-seq data

To classify individual cells as originating from responder or non-responder samples, we implemented two deep learning architectures using Keras: a 1D-CNN and a two-dimensional convolutional neural network (2D-CNN). For the pathway-based model, we downloaded KEGG pathways (186 gene sets) from MSigDB (https://www.gsea-msigdb.org/gsea/msigdb). To generate the input file for the 1D-CNN model, we linked individual genes to their corresponding pathways. After removing duplicate gene–pathway combinations, we constructed expression matrices for a total of 12,413 unique gene–pathway pairs across 16,290 single cells (S1B Fig). Meanwhile, in the pathway-level modeling, the two types of information (gene names and pathway names) enabled a 2D data representation, allowing us to apply a 2D-CNN; accordingly, a 2D-CNN model was constructed. For the 2D-CNN model, we generated two-by-two matrix files for each of the 16,290 single cells, where each matrix consisted of gene expression values with 186 pathways represented as columns and 383 genes in the pathways as rows (S2 Fig in S1 File). Given that the input data for the pathway-based model were limited to 383 genes, the model was constructed without an additional feature selection step.

The 1D-CNN model received a flattened vector of gene-pathway features per cell with a shape of (12,413, 1). The flattened vector comprised three sequential convolutional blocks: the first block included 128 filters with a kernel size of 15, followed by ReLU activation, dropout (rate = 0.3), and max pooling (pool size = 2). The second block used 64 filters with the same kernel size and operations, and the third block used 32 filters with a kernel size of 5. The resulting feature maps were flattened and passed through a fully connected dense layer with 64 units and ReLU activation, followed by dropout and a final sigmoid-activated output layer for binary classification.

In parallel, the 2D-CNN model leveraged the spatial structure of the gene-pathway matrix. Each cell was represented as a grayscale image of size 383 × 186 (genes × pathways), with input shape (383, 186, and 1). The architecture consisted of two convolutional blocks: the first with 128 filters (3 × 3 kernel, ‘same’ padding), and the second with 64 filters (3 × 3 kernel), both followed by ReLU activation, dropout (rate = 0.3), and 2 × 2 max pooling. A global max pooling layer was then applied, followed by a dense layer with 64 units, ReLU activation, and dropout, culminating in a sigmoid-activated output layer.

Both models were trained using the Adam optimizer (learning rate = 1e-4) and binary cross-entropy loss. Early stopping (patience = 3) and model checkpointing based on validation accuracy were employed to mitigate overfitting. The 1D-CNN was trained with a batch size of 64 for up to 30 epochs using normalized input features, while the 2D-CNN followed the same training protocol using the reshaped 2D inputs. All models were trained using identical train/validation splits to ensure a fair comparison.

All implementations were developed using Keras (v2.8.0) with TensorFlow (v2.8.0) as the backend. To interpret the predictions of the 2D-CNN model, we applied Gradient-weighted Class Activation Mapping (Grad-CAM) to visualize the most influential gene-pathway regions. Grad-CAM was implemented using GradientTape from TensorFlow for automatic differentiation. Grad-CAM derives class-discriminative importance scores from the activation maps of the final convolutional layer, weighted by the gradients of the predicted class score. Accordingly, the resulting heatmaps represent activation-based contribution patterns learned by the 2D-CNN, rather than absolute gene expression levels. Color intensity reflects the relative importance of convolutional features associated with specific gene–pathway regions for the model’s prediction.

Specifically, we constructed a sub-model that outputs both the feature maps from the final convolutional layer and the model prediction. For a given input cell, the gradient of the predicted class score with respect to the final convolutional feature maps was computed using GradientTape. These gradients were spatially average-pooled to obtain importance weights for each feature map channel, which were then linearly combined with the feature maps to generate a class-discriminative heatmap. The resulting map highlights gene-pathway regions that most strongly contributed to the decision of the model. The performance of the proposed deep learning models (1D-CNN and 2D-CNN) was evaluated based on their ability to classify single cells according to patient response to ICI therapy (responder vs. non-responder). The ROC-AUC was used as the primary evaluation metric, given its robustness in binary classification tasks, especially under potential class imbalance.

To contextualize the performance of the CNN-based models, a series of conventional machine learning classifiers was also implemented as baselines. These included logistic regression, SVM, Random Forest, extreme gradient boosting (XGBoost), and feedforward neural network (FNN). All baseline models were trained on the same input data as the 1D-CNN (flattened gene-pathway feature vectors). Hyperparameters for each model were optimized using grid search or default configurations provided by standard machine learning libraries such as scikit-learn and XGBoost. The CNN and baseline models were trained and validated on identical data splits to ensure a fair comparison.

### Bulk RNA-seq data analysis for AI predictive model validation

For validation purposes, bulk-RNA dataset of melanoma (Liu dataset) was also downloaded from cBioPortal (https://www.cbioportal.org) [27,28]. This dataset includes tumor tissue samples from 144 patients with advanced melanoma treated with anti-PD1 immune checkpoint blockage monotherapy, among whom RNA-seq data were available for 121 patients. Among them, 47 patients were classified as responders, including those with a complete response (CR) or partial response (PR) to ICI, whereas 72 non-responders exhibited progressive disease (PD) or stable disease (SD). Two patients exhibited heterogeneous treatment responses.

In addition, we downloaded raw RNA-seq FASTQ files from melanoma patient tissues treated with either anti-PD-1 monotherapy or combined anti-PD-1 and anti-CTLA-4 therapy (https://www.ebi.ac.uk/ena/browser/home; accession number PRJEB23709) (Gide dataset) [29]. Gene expression values were quantified using the Cancer Genome Atlas Program (TCGA) RSEM RNA sequencing analysis pipeline and normalized using the upper quartile normalization method [30]. Gene expression level was further transformed by log2(expression value +1) prior to downstream analyses. Because *MTRNR2L2* was not included in the hg19 based reference gene annotation used by this pipeline, expression values were unavailable. The function of this gene has been elucidated only recently, and therefore this gene was not included in the annotation of RNA-seq analysis pipelines based on early versions of the hg19 reference genome. Data regarding the transcriptome-based molecular subtypes [31] for this dataset were retrieved from a study conducted by Bagaev et al. [32].

We also downloaded 108 bulk RNA-seq datasets (57 on-treatment and 51 pre-treatment) from 65 patients with melanoma, along with the corresponding clinical data, from the GEO database (GSE91061, Riaz dataset) [33]. The clinical characteristics and RNA sequencing platform information for the three independent datasets are summarized in S1 and S2 Tables in S1 File, respectively.

### Signature scoring for gene set using bulk RNA-seq data

Signature scores were calculated using the singscore package (v1.19.1) [34] based on gene lists derived from a 2D-CNN prediction model using the KEGG pathway. These signature scores were applied to three independent melanoma bulk RNA-seq datasets from ICI-treated patients.

### Statistical analysis

Continuous variables, such as gene expression and immune exhaustion score, were compared using the Wilcoxon rank sum test. Survival analysis was performed using the log-rank test, with visualization using Kaplan–Meier plots. Additionally, univariate or multivariate Cox proportional hazards regression analyses were performed using R (v4.1.2). Forest plots were visualized using the *survminer* R package (v0.5.0). Survival was analyzed with the survival R package (v3.2-13). All P values reported are two-sided. All statistical analyses were performed with R (v4.1.2) or Python (v3.10.12).

## Results

### Malignant melanoma TME landscape and immuno-therapy response marker identification

**Fig 1:**
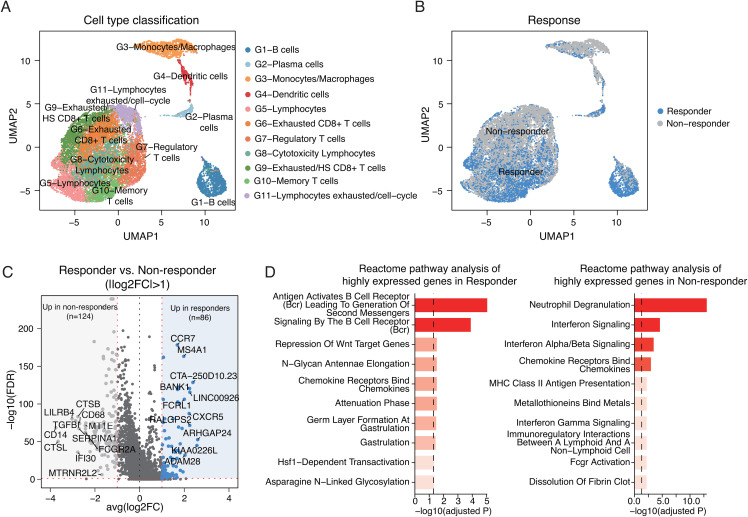
scRNA-seq characteristics of metastatic melanoma by immunotherapy response. **(A)** UMAP visualization of cell types in melanoma tissues. **(B)** UMAP plot illustrating the distribution of immunotherapy responders and non-responders. Responders (blue) and non-responders (gray) exhibit distinct clustering patterns. **(C)** Volcano plot showing differentially expressed genes (DEGs) between responders (n = 86) and non-responders (n = 124) identified across all cells regardless of cell type from scRNA-seq data (|avg log2FC| > 1). Top 10 genes based on log2FC in responders and non-responders are labeled. **(D)** Reactome pathway analysis of highly expressed genes identified from scRNA-seq data in responders (left) and non-responders (right). scRNA-seq, single cell RNA sequencing; FC, fold change.

Using the metastatic melanoma scRNA-seq dataset (GSE120575), we obtained a raw UMI matrix of melanoma tissues. After filtration, the expression profiles of 16,290 cells were processed using Seurat R package. UMAP and previously published cell type information revealed distinct cell types, including T lymphocytes and myeloid, B, and plasma cells (**Fig 1A**). These cell types were identified based on the expression of canonical markers, such as CD3D and CD3E, in T lymphocytes [24]. Additionally, when classified by immunotherapy responsiveness, differences in cell composition and the proportion of specific cell types were observed (**Fig 1B** and S3 Fig in S1 File). To investigate the difference in transcriptional profiles between responders and non-responders independent of cell types, DEGs were identified between the two groups. This analysis identified 210 DEGs between responders and non-responders, including 86 genes upregulated in responders and 124 genes upregulated in non-responders (**Fig 1C**). In the non-responder group, gene expression was enriched for pathways related to neutrophil degradation, interferon signaling, interferon-gamma signaling, and MHC class II antigen presentation. In contrast, the responder group exhibited activation of pathways associated with B cell response and B cell receptor signaling (**Fig 1D**).

### Key gene contributors identified by feature selection from gene-based prediction model using identified DEGs of scRNA-seq data

**Fig 2:**
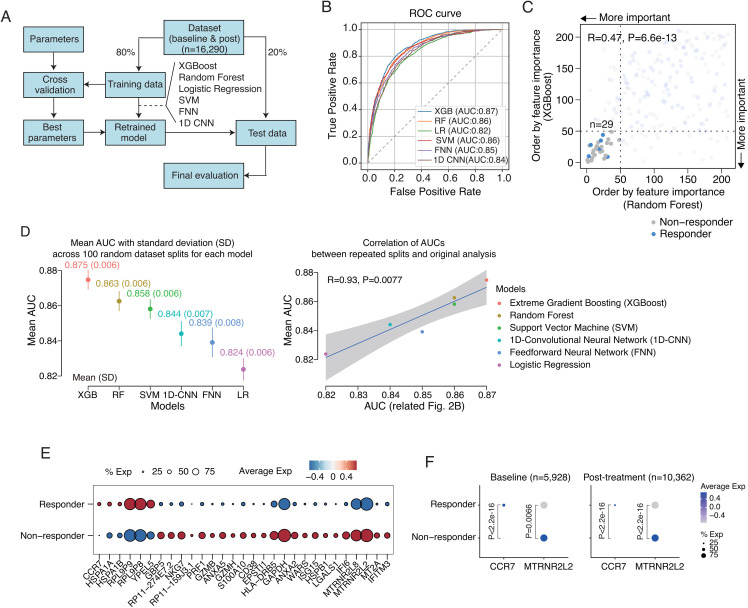
Gene-based classification model using scRNA-seq data for immunotherapy response. **(A)** Schematic overview of classification model building using differentially expressed genes (DEGs). Dataset (n = 16,290; baseline and post-treatment) was split into 80% training and 20% test sets. Various models, including XGBoost, Random Forest, Logistic Regression, SVM, FNN, and 1D CNN, were trained using the training set. Hyperparameters were optimized through cross-validation and the best parameters were used to retrain the models. The retrained models were then evaluated on testing set to obtain final performance metrics. **(B)** ROC curves of classification models. XGBoost achieved the highest area under the curve (AUC = 0.87), followed by Random Forest (AUC = 0.86) and SVM (AUC = 0.86). **(C)** Scatter plot comparing feature importance rankings from XGBoost and Random Forest. A subset of 29 genes was consistently identified as important for classification. **(D)** To assess the reproducibility of the predictive model, we permuted the dataset 100 times and measured AUC of the model; the results were similar to the original test (Spearman correlation test). **(E)** Dot plot showing expression levels of 29 genes in responders and non-responders (related to **Fig 2C**). **(F)** Differences in CCR7 and MTRNR2L2 expression between responders and non-responders in baseline and post-treatment samples, respectively (Wilcoxon rank-sum test). %Exp indicates the percentage of cells expressing the gene.

Six classification models were built based on the 210 DEGs selected through feature selection to predict responders and non-responders (**Fig 2A** and S1A Fig in S1 File). In this study, XGBoost, Random Forest, logistic regression, SVM, FNN, and 1D CNN were trained and evaluated. Among these models, XGBoost achieved the highest performance with an area under the ROC-AUC of 0.87, followed by Random Forest (AUC: 0.86) and SVM (AUC: 0.86) (**Fig 2B**). In contrast, logistic regression (AUC: 0.82) and 1D-CNN (AUC:0.84) showed the lowest performance among the models. To identify the most influential genes with high feature importance in tree-based models with the highest performance, we compared feature importance rankings derived from XGBoost and Random Forest. A significant correlation (R = 0.47, P = 6.6e-13) was observed between the rankings derived from both tree-based methods, highlighting a subset of 29 genes as consistently important for prediction (**Fig 2C**). As a supplementary assessment of reproducibility, we performed 100 independent random splits of the dataset and repeatedly computed the AUC to derive confidence intervals (**Fig 2D**). The resampled estimates showed a strong correlation with the original test results (R = 0.93, P = 0.0077), and XGBoost consistently achieved the highest predictive performance.

Expression of these key 29 genes showed distinct patterns between responder and non-responder groups (**Fig 2E**, S4 Fig, and S3 Table in S1 File). Among these genes, analysis of their association with post-treatment survival using the Liu dataset revealed that *CCR7* and *MTRNR2L2* were significantly correlated with patient survival. Accordingly, we focused our subsequent analyses on these two genes. Notably, when baseline and post-treatment scRNA-seq samples were analyzed separately, these genes remained significant regardless of treatment time point (**Fig 2F**).

### *CCR7* and *MTRNR2L2* expression patterns across immune cell types derived from scRNA-seq data

**Fig 3:**
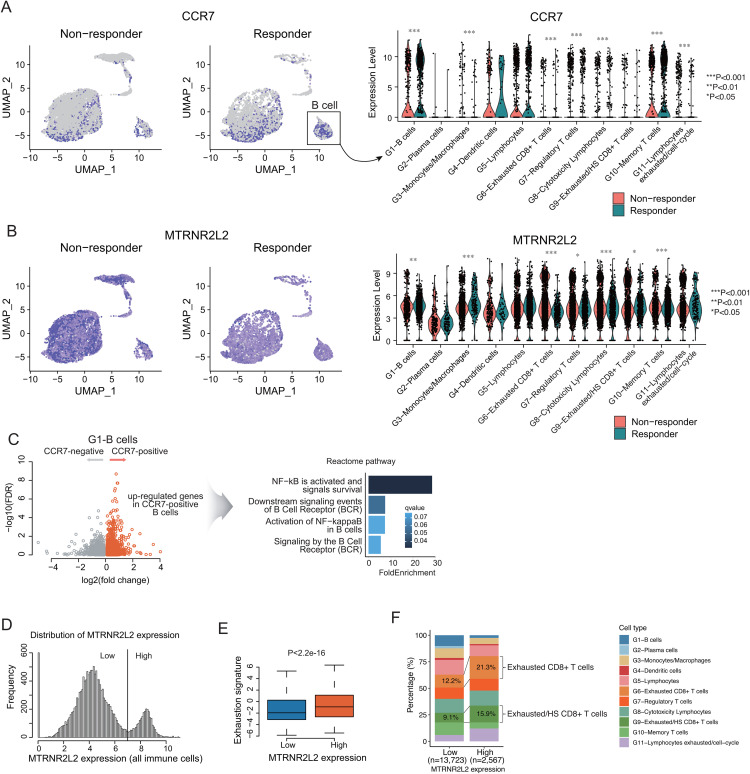
Expression patterns of *CCR7* and *MTRNR2L2* in scRNA-seq data. **(A)** *CCR7* is predominantly expressed in B cells and memory T cells within the responder group (Wilcoxon rank-sum test)*.* **(B)** *MTRNR2L2* was broadly expressed across multiple cell types; however, its expression was particularly elevated in exhausted CD8 ⁺ T cells within the non-responder group (Wilcoxon rank-sum test). **(C)** Pathway enrichment analysis of differentially expressed genes between CCR7-positive and CCR7-negative cells within the G1-B cell cluster revealed enrichment of B cell activation-related pathways, including NF-κB signaling, in CCR7-positive B cells. **(D)** Biomodal distribution pattern of *MTRNR2L2* gene expression. **(E)** High *MTRNR2L2* expression was associated with elevated immune exhaustion scores (Wilcoxon rank-sum test). **(F)** Proportion of exhausted CD8 ⁺ T cells increased in high-MTRNR2L2 expression group.

Using cell type annotation information from the scRNA-seq data, we examined the expression patterns of the *CCR7* and *MTRNR2L2* genes. *CCR7* showed high expression in the responder group, consistent with its identification in the prediction model, and was particularly enriched in B cells and memory T cells (**Fig 3A**). In contrast, *MTRNR2L2* exhibited overall high expression in the non-responder group, with elevated expression observed across various T cell populations, including exhausted CD8 ⁺ T cells and cytotoxic lymphocytes (**Fig 3B**). The remaining 27 genes are presented in S5 Fig in S1 File. Functionally, pathway analysis revealed that CCR7-positive B cells showed enrichment of immune activation-related pathways, such as NF-κB signaling, compared to CCR7-negative B cells (**Fig 3C**). *MTRNR2L2* expression displayed a distinct bimodal distribution pattern (**Fig 3D**), with the high-expression group exhibiting a significantly elevated immune exhaustion score (**Fig 3E**). Cells with high *MTRNR2L2* expression exhibited a higher proportion of exhausted CD8 ⁺ T cells than low-expression cells (**Fig 3F**).

The functions of other key genes identified in our analysis (*IFITM3*, *MT2*, *MTRNR2L8*, *IFI6*, *HSPA1A*, *HSPA1B*, *RPL9P9*, and *RPL9P8*) in the immune system and cancer are summarized in S4 Table in S1 File [35–47]. Several of these genes participate in inflammatory responses within the immune microenvironment and are implicated in tumor growth and oncogenesis. For example, *IFITM3* exhibits dual functionality, contributing to antiviral defense and promoting tumorigenesis [35–37,47]. *HSPA1A* and *HSPA1B*, members of the heat-shock protein family, can augment immune activation; however, polymorphisms in these genes have been associated with increased melanoma risk [46]. *MT2A* is overexpressed in melanoma and has been implicated in downregulating adaptive immunity [38,39].

### *CCR7* and *MTRNR2L2* expression validation using independent bulk RNA-seq cohorts

**Fig 4:**
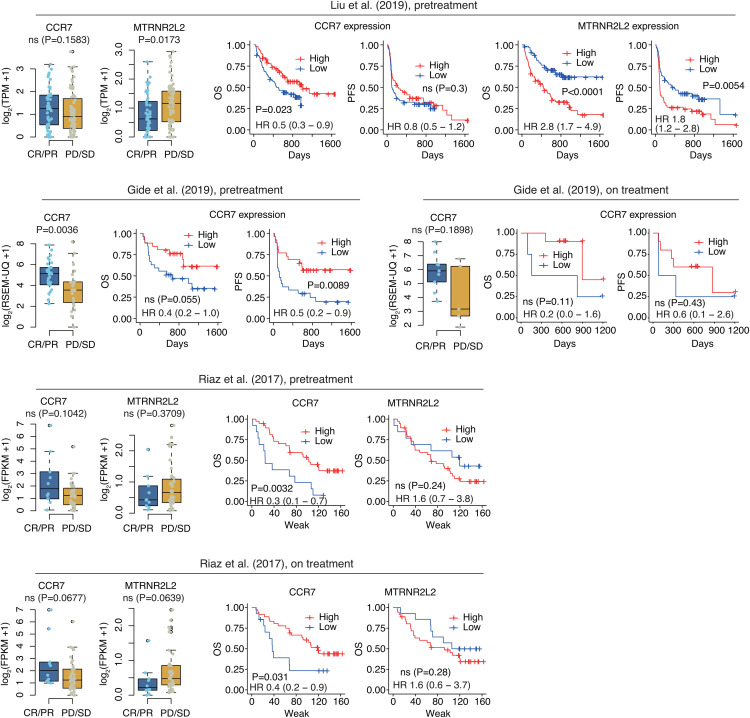
Clinical significance of CCR7 and MTRNR2L2 across three independent melanoma cohorts with bulk RNA-seq data. Box plots displaying the expression of genes (*CCR7* and *MTRNR2L2*) in three independent bulk RNA-seq data from melanoma patients with CR/PR (complete response/partial response) and PD/SD (progressive disease/stable disease). Kaplan–Meier survival curves demonstrating associations of genes with overall survival (OS) and progression-free survival (PFS) (log-rank test). Hazard ratio (HR) and 95% confidence intervals from the univariate Cox regression analysis. High *CCR7* expression is associated with improved survival, whereas high *MTRNR2L2* expression is associated with worse prognosis.

We examined the association between *CCR7* and *MTRNR2L2* expression and immunotherapy response and prognosis in three independent cohorts of melanoma patients treated with immune checkpoint inhibitors (ICIs) (**Fig 4**). In the Liu dataset, *CCR7* expression was associated with favorable survival, whereas *MTRNR2L2* expression was associated with unfavorable survival. These findings were further validated using two independent datasets (the Gide and Riaz datasets). Although some associations did not reach statistical significance, consistent trends were observed across all datasets. In summary, across all three datasets, *CCR7* tended to be highly expressed in patients with favorable treatment responses, and higher *CCR7* expression showed a tendency to be associated with improved survival. In contrast, *MTRNR2L2* exhibited the opposite trend. Similar patterns were observed in both pre-treatment and on-treatment samples. The expression patterns of the remaining key genes identified by the predictive model, stratified by treatment response, are presented in S6 Fig in S1 File.

### Prognostic significance of *CCR7* and *MTRNR2L2* according to histopathologic and molecular subtypes

**Fig 5:**
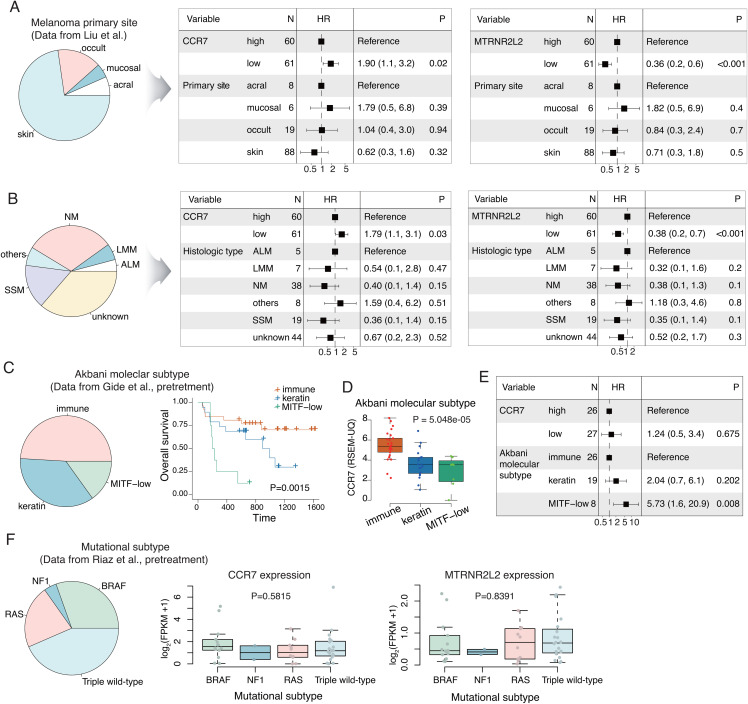
*CCR7* and *MTRNR2L2* expression according to the melanoma subtypes. **(A, B)** *CCR7* and *MTRNR2L2* expression showed prognostic significance for overall survival, independent of the primary site (A) and histological subtype of melanoma **(B). (C)** Prognostic differences according to molecular subtypes, including microphthalmia-associated transcription factor (MITF)-low, immune, and keratin subtypes (log-rank test). **(D)** *CCR7* expression patterns across molecular subtypes (Kruskal–Wallis test). **(E)** Multivariate Cox regression analysis of *CCR7* expression in relation to molecular subtype. ALM, acral lentiginous melanoma; LMM, lentigo malignant melanoma; NM, nodular melanoma; SSM, superficial spreading melanoma. **(F)** Gene expression patterns across mutational subtypes (Kruskal–Wallis test).

The three melanoma cohorts used for validation consisted of melanomas from various primary sites. Using these cohorts, we investigated the prognostic significance of *CCR7* and *MTRNR2L2* genes across histopathological and molecular subtypes. The prognostic significance of *CCR7* and *MTRNR2L2* expression was consistent regardless of the primary site (**Fig 5A**) or histologic subtype (**Fig 5B**) of melanoma. In contrast, when examining the three molecular subtypes of cutaneous melanoma, the immune subtype was associated with the most favorable prognosis, whereas the MITF-low subtype showed the poorest prognosis (**Fig 5C**). *CCR7* gene expression was significantly associated with molecular subtype, showing markedly higher expression in the immune subtype (**Fig 5D**). The prognostic significance of *CCR7* expression was lost in multivariate analysis that included molecular subtype (**Fig 5E**). When we examined the mutational subtypes using the Riaz dataset [33], *CCR7* and *MTRNR2L2* expression showed no association with mutational subtype (**Fig 5F**).

### Pathway-based deep learning approach for predicting immunotherapy response

**Fig 6:**
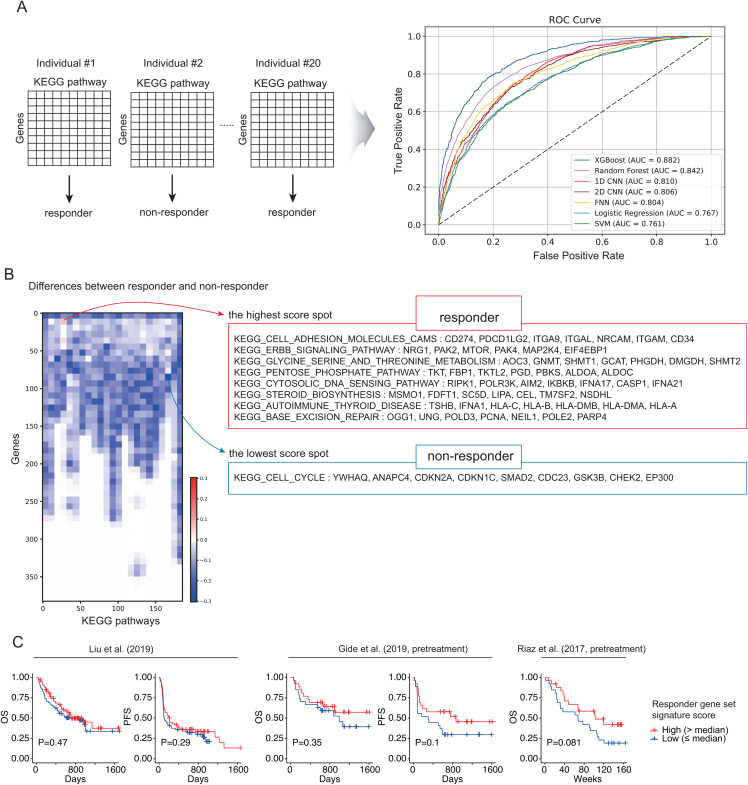
Pathway-based classification models based on the KEGG pathway. **(A)** Single-cell gene expression mapped to KEGG pathways enabled predictive models showing relatively high performance across multiple AI algorithms. **(B)** Heatmap (left) showing differences in 2D-CNN feature-importance between responders and non-responders; numbers on x- and y-axes denote distinct genes and pathways, respectively. List of pathways and their constituent genes showing the largest differences between responders and non-responders (right panel). **(C)** Prognostic analysis across three bulk RNA-seq datasets using responder-specific signature scores derived from genes within the eight responder-associated pathways identified in the highest score spot in Fig 6B (log-rank test).

Although individual genes—such as *CCR7* and *MTRNR2L2*—showed associations with ICI response and prognosis of patients, we examined whether prediction was possible at the pathway level. KEGG pathway-level analysis showed varying predictive performance across different classification models (**Fig 6A**). In the pathway-level approach, XGBoost (AUC: 0.882) and Random Forest (AUC: 0.842) showed the highest predictive performance, followed by 1D-CNN (AUC: 0.810), 2D-CNN (AUC:0.806), FNN (AUC: 0.804), Logistic Regression (AUC: 0.767), and SVM (AUC: 0.761). To identify and visualize the contribution of individual gene–pathway combinations to ICI response prediction in the 2D-CNN model, we applied the Grad-CAM technique. For both responder and non-responder groups, we generated heatmaps with genes on the x-axis and pathways on the y-axis, where higher values indicate gene–pathway combinations that contributed most to model prediction within each response category (S7 Fig in S1 File). This visualization enabled us to distinguish gene–pathway associations specific to either responders or non-responders. Accordingly, the feature importance analysis of the 2D-CNN model revealed key gene–pathway features that contributed to the prediction of response and non-response (S7 Fig in S1 File). When the differences in gene–pathway feature values between responders and non-responders were visualized using a heatmap (**Fig 6B**), the two spots showing the largest positive and negative differences each encompassed 64 genes across eight pathways, suggesting that these features represent the most critical elements characterizing responders and non-responders, respectively. This region included a substantial number of immune-related genes, such as immune checkpoint gene *CD274*, *HLA* genes, and interferon-associated genes. Conversely, the corresponding region in non-responders primarily involved nine genes belonging to the cell cycle pathway (Fig 6B). The *CCR7* gene, which was identified at the single-gene level, was not ranked among the top features in the pathway-level prediction model. Meanwhile, when responder-specific signature scores derived from genes in these pathways from the highest score spot **(**Fig 6B**, red box)** were calculated across three independent bulk RNA-seq datasets, no significant differences in prognosis were observed, although a trend toward improved survival was noted (Fig 6C).

## Discussion

In this study, we developed a predictive model to classify immunotherapy responders and non-responders using scRNA-seq data without requiring prior cell type annotation. Model utility was evaluated at both the single-gene and the pathway levels. Apart from XGBoost, the overall performance of the classification models using pathway-derived features was lower than models built with DEG-based features, suggesting that individual DEGs may provide more informative signals for ICI response prediction than aggregated pathway-derived features. The single-gene-based model identified *CCR7* and *MTRNR2L2* as the most influential genes for predicting responders and non-responders, respectively, validated in an independent large-scale cohort.

*CCR7* is a key homing receptor and regulator of antigen-specific immune response [43,44]. Consistent with our findings, *CCR7* is associated with a favorable prognosis in melanoma [44]. In our study, differential gene expression analysis between responders and non-responders revealed an upregulation of B cell receptor signaling in the responder group. Notably, *CCR7* was also highly expressed in B cells from responders. *MTRNR2L2* is a key gene regulated by TET3-mediated DNA-demethylation [41]. Glyphosate exposure induces *MTRNR2L2* demethylation, an effect that persists even after exposure stops. These epigenetic features suggest its potential as a biomarker for long-term changes in the TME. Since ICI responsiveness is closely related to the TME, high *MTRNR2L2* expression is associated with non-responders and poor prognosis, indicating its role in ICI resistance.

Several additional key genes identified in our study (S3 Table in S1 File) are linked to immunity and tumorigenesis. *IFITM3* overexpression promotes tumor growth and metastasis and increases the expression of immunosuppressive checkpoint genes, thereby dampening antitumor immunity and facilitating immune evasion [48]. By contrast, recent studies suggest that *IFITM3* can serve as a marker of immune-hot tumors across a broad range of cancers and may potentially enhance responses to ICIs by augmenting MHC-I expression [49,50]. These seemingly divergent findings likely reflect context-dependent, cell type-specific, and pathway-specific differences in *IFITM3* downstream signaling [48]. *MT2A* is overexpressed in melanoma and may reduce adaptive immunity, thereby increasing tumor immune evasion [38,51]. In addition, heat-shock proteins play a prominent role in tumor progression and drug resistance; by facilitating tumor adaptation to diverse microenvironmental stressors—such as hypoxia, immune pressure, and nutrient deprivation—they contribute to HSP-mediated resistance to anticancer therapy [52].

In our pathway-based model, several key pathways were associated with ICI response. Cell cycle-related pathways were enriched in responders, whereas non-responders showed enrichment in pathways, such as cell adhesion, erb-b2 receptor tyrosine kinase (ERBB) signaling, glycine/serine/threonine metabolism, pentose-phosphate, cytosolic DNA sensing, steroid biosynthesis, autoimmune thyroid disease, and base excision repair (BER) pathways. Cell cycle alterations may contribute to immune evasion mechanisms of tumor-infiltrating immune cells. G1/S checkpoint arrest, mediated by cell cycle inhibitors, can reduce immune cell responsiveness to tumors, maintaining an immunosuppressive state [53]. Conversely, activation of proliferative factors such as CDK4/6 is associated with immune cell exclusion and poor response to ICIs [53]. The ERBB signaling pathway influences immunotherapy efficacy by upregulating PD-L1 expression within the TME [54]. In our study, in addition to ERBB signaling, we also observed upregulation of PD-L1, which is annotated in the KEGG “cell adhesion molecules” pathway, further supporting an immunomodulatory role for ERBB-related signaling. Tumors with high serine/glycine metabolism exhibit increased PD-1/PD-L1 expression and cytotoxic T cell infiltration, associated with improved ICI response [55]. The cyclic GMP-AMP synthase–stimulator of interferon genes (cGAS–STING) pathway, a central component of the cytosolic DNA sensing pathway, plays a pivotal role as an innate immune sensor of cytosolic DNA and significantly shapes the tumor–immune interface and response to immunotherapy [56]. STING activation leads to the production of type I interferons and chemokines (e.g., CXCL9, CXCL10, and CCL5) that facilitate T cell recruitment to the TME, thereby enhancing anti-tumor immunity [56]. Lastly, BER deficiencies are associated with increased tumor immunogenicity, greater neoantigen burden, and elevated PD-L1 expression, suggesting that BER status may serve as a predictive biomarker for favorable ICI responses [57].

In this study, important features identified by the single gene-based prediction model, such as *CCR7* and *MTRNR2L2*, were not selected in the pathway-level prediction model. Models based on individual genes and those based on pathways each have their own advantages and limitations. The lack of full concordance between the two approaches may arise from several factors. One possible explanation is that within a given pathway, some genes may exhibit positive associations while others show negative associations (S8 Fig in S1 File), leading to a reduction in overall importance when aggregated at the pathway level. Therefore, designing predictive models that take such characteristics into account, depending on the intended purpose of the analysis, is necessary.

In pathway-based AI analyses, the results depend on which pathway dataset is utilized. In this study, we used the KEGG pathway database because the cells constituting the TME are all non-neoplastic, and KEGG can encompass pathways not only related to diseases but also those present in normal cells. Moreover, KEGG pathways comprehensively cover both gene expression and metabolic processes, which makes them particularly suitable for our analysis because recent studies have highlighted that metabolic reprogramming within the TME plays a critical role in enhancing responses to immunotherapy [58].

Although our study does not claim that an annotation-free framework outperforms label-based analyses, it offers complementary value. Because it operates without cell labels, it avoids potential label bias and enables de novo discovery of cross-cell programs directly linked to clinical response. In our data, features prioritized by the label-free screen (e.g., CCR7 and MTRNR2L2) can be retrospectively localized to specific immune subsets when inspected with annotations, thereby facilitating biological interpretation. Notably, CCR7 showed consistent associations with ICI responsiveness and clinical outcomes in independent melanoma datasets, and our literature review supports its mechanistic rationale. Conclusively, these validation layers indicate that an annotation-free framework can perform robustly in this setting. In line with our findings, a previous study trained an AI model on all immune cells using scRNA-seq data to predict immunotherapy response, and identified *CCR7* as a key predictive gene [59].

Differences in cellular populations between responder and non-responder groups have been well documented [60]. Accordingly, variations in gene expression may originate from such differences in cell populations, and it is plausible that our AI model, trained without cell-type annotation, has captured these gene expression differences driven by cellular heterogeneity. In the case of MTRNR2L2, its expression was observed across multiple cell clusters that constitute a large proportion of the total cell population. This suggests that differences in expression may reflect disparities in cell composition and variation in expression levels within the same cellular subtype, warranting careful interpretation. In an additional validation dataset, CCR7 was consistently associated with treatment response, whereas MTRNR2L2 did not reach statistical significance, indicating that further validation may be required.

In contrast, label-based analyses are clear benefits to simplifying the high-dimensional structure of single-cell sequencing data or leveraging per-cell annotations [19,20], a key advantage of annotation-free models is their capacity to discover novel patterns or previously unrecognized subtypes beyond predefined cell types. By analyzing the entire cell population, such models can capture broader patterns and avoid errors that may arise during cell type annotation. In particular, cell type annotation in scRNA-seq data is not yet standardized and often varies significantly across studies, with annotation errors remaining common. Therefore, omitting annotation may actually enhance the robustness and scalability of AI model development, especially when integrating large-scale datasets generated by different researchers.

Among the models evaluated in this study, XGBoost consistently outperformed the AI models. This finding is likely related to the inherently high-dimensional and sparse nature of scRNA-seq data. As a tree-based ensemble method, XGBoost can effectively handle sparsity through built-in regularization while capturing nonlinear interactions among genes. In contrast to deep learning models that generally require large datasets for robust generalization, XGBoost can achieve stable performance even with relatively small cohorts, making it particularly suitable for single-cell studies where sample sizes are often limited. Such trade-offs between model characteristics and performance have also been consistently reported in recent benchmark studies, which demonstrate that the relative performance of annotation methods depends strongly on factors such as dataset size, rare-cell prevalence, and batch-effect magnitude [61].

Beyond the scope of this study, other modeling approaches may also represent promising alternatives. For example, scVI, a variational autoencoder-based framework, simultaneously models sparsity and batch effects in scRNA-seq data while learning low-dimensional latent representations that enable diverse downstream analyses, such as cell annotation [62]. Similarly, scGNN, a graph neural network-based model, explicitly incorporates relational information such as protein–protein interactions and cell–cell similarity to infer biologically meaningful representations, and has demonstrated superior performance in tasks including clustering and gene expression imputation [63]. Recently, hybrid architectures such as scGraPhT, which integrate Transformers with GNNs, have been proposed to capture both gene- and cell-level relationships while leveraging pretrained representations for efficient annotation [64].

Taken together, model selection should be considered not solely on the basis of predictive accuracy, but also in the context of dataset characteristics, cohort size, interpretability, and computational resources. The superior performance of XGBoost in our analysis can thus be understood as part of this broader landscape of trade-offs, while future work integrating GNN- or autoencoder-based approaches may further enhance both the predictive power and biological interpretability of single-cell analyses.

Despite its potential for broader application, this study has several limitations and raises ethical considerations. First, our study relies on scRNA-seq data, which is not typically obtainable from formalin-fixed paraffin-embedded (FFPE) samples, the standard clinical pathology. Given that FFPE is the most commonly used form in clinical settings, validating the key genes identified by our AI-based prediction framework using bulk RNA-seq data derived from FFPE samples is crucial. Therefore, in future work, we intend to explore the feasibility of developing an FFPE-optimized multi-gene PCR panel, including *CCR7* and *MTRNR2L2*, based on the gene combinations identified in this study, and to evaluate its predictive performance in a retrospective cohort. Second, while our conclusions are supported across independent transcriptomic cohorts, protein-level validation is essential as post-translational modification and proteasomal degradation may alter functional expression. We also clarify that our current models are trained on transcript-level features and do not assume proportional mapping to protein abundance. Therefore, validation at the protein level will also be necessary. Furthermore, while our annotation-free DEG approach demonstrated robust performance in transcriptomic datasets, its generalizability and applicability to other high-dimensional data modalities, such as proteomics or spatial transcriptomics, warrant further validation. In addition, experimental validation to understanding how *MTRNR2L2* regulates ICI responsiveness remains lacking. Therefore, further studies should focus on functional experiments to clarify these mechanisms. Third, although neutrophils and neutrophil extracellular trap formation (NETosis) are important in melanoma metastasis [65], neutrophils are notoriously under-captured in scRNA-seq [66,67]. In our analysis dataset, neutrophils were essentially absent. Consequently, genes related to neutrophil biology or NETosis could not be leveraged by the AI model, which represents a limitation of this study. Fourth, public datasets are widely used to develop AI models, including those used in genomics. Even when analyses rely on anonymized public data, clinical translation still poses ethical risks, dual use/malicious repurposing, overreliance aftereffects, and omission risks from underrepresented groups. There are also limitations in performing integrated analyses due to differences in gene expression units across datasets.

**Fig 7:**
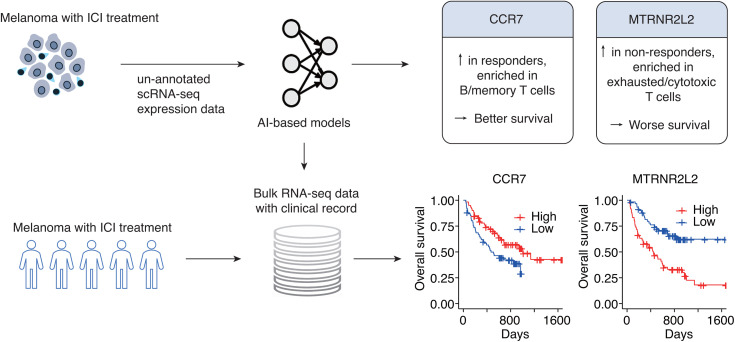
Summary of results. AI-based identification of immunotherapy response markers from single-cell RNA-seq without cell type annotation, validated in independent bulk RNA-seq cohorts.

In conclusion, we demonstrate that predictive markers associated with ICI treatment response can be identified by building a prediction model directly from scRNA-seq data without cell type annotation (Fig 7). This highlights the potential of annotation-free methods to uncover biologically relevant patterns and support clinically practical predictions using a limited set of genes.

## Supporting information

S1 Fig Input data format. (A) Input format of single-cell RNA-seq data for single gene-based modeling using six models. All six models used a same cell × DEG matrix (cell-by-gene matrix) as their input. Specifically, each row represents an individual cell and each column corresponds to selected differentially expressed genes (DEGs), and this gene expression matrix was used as a common input across all models. (B) Input format of single-cell RNA-seq data for pathway-based modeling using 1D-CNN, XGBoost, Random Forest, feed-forward neural network (FNN), logistic regression, and SVM. In this study, we collected 186 pathways and their corresponding gene sets from the KEGG database. Each gene-pathway combination was treated as a distinct feature, resulting in the construction of a cell-by-gene–pathway pair expression matrix. For example, if gene A belongs to pathway I, a column labeled “geneA.PathwayI” was created. Similarly, if both gene A and gene B belong to pathway II, separate features such as “geneA.PathwayII” and “geneB.PathwayII” were generated. Through this approach, we obtained a total of 12,413 unique gene–pathway pair features, where each row represents a single cell and each column represents a specific feature. The matrix values correspond to log₂-transformed TPM values, indicating the expression level of a specific gene within a specific pathway in each cell. Therefore, the numbers presented in the matrix represent the log₂-transformed TPM expression values for the corresponding cells (rows).
(DOCX)

S1 File **S2 Fig.** Input format of single-cell RNA sequencing data for pathway-based modeling using the 2D Convolutional Neural Network (2D CNN) model. **S3 Fig.** Differences in immune cell profiles according to immunotherapy response in single-cell RNA-seq data from malignant melanoma patients treated with immunotherapy. **S4 Fig**. Expression patterns of top contributing genes from the scRNA-seq based predictive model, stratified by immunotherapy response group (responders vs. non-responders). **S5 Fig.** Expression patterns of 29 genes across immune cell types derived from scRNA-seq data. **S6 Fig**. Gene expression pattern of other key genes according to treatment response across three independent melanoma cohorts with bulk RNA-seq data (Wilcoxon rank-sum test). **S7 Fig**. Feature importance heatmap of responders and non-responders, derived from a 2D Convolutional Neural Network (2D-CNN) model using KEGG pathways. **S8 Fig**. Expression patterns of genes within the KEGG Chemokine Signaling Pathway (including CCR7) in responders versus non-responders. Even within the same pathway, several genes, such as CCR7, show high expression in responders, whereas others are higher in the non-responders, highlighting intra-pathway heterogeneity. **S1 Table.** Pathological and clinical characteristics of three distinct melanoma cohorts treated with immunotherapy analyzed in this study. **S2 Table.** RNA sequencing platforms used in the three distinct melanoma cohorts. **S3 Table.** Summary statistics of expression for 29 genes in responders and non-responders. **S4 Table**. Top 10 genes identified by our AI model in relation to immunotherapy responder and non-responders in melanoma.
(ZIP)
