# Supplementary material for: Annotation-free prediction of immunotherapy response in melanoma using single-cell transcriptomic data
Source: PLoS One. 2026 Feb 27;21(2):e0343633. doi: 10.1371/journal.pone.0343633 (PMC12948085; doi:10.1371/journal.pone.0343633)
Supplement: S1 Fig — (A) Input format of single-cell RNA-seq data for single gene-based modeling using six models. All six models used a same cell × DEG matrix (cell-by-gene matrix) as their input. Specifically, each row represents an individual cell and each column corresponds to selected differentially expressed genes (DEGs), and this gene expression matrix was used as a common input across all models. (B) Input format of single-cell RNA-seq data for pathway-based modeling using 1D-CNN, XGBoost, Random Forest, feed-forward neural network (FNN), logistic regression, and SVM. In this study, we collected 186 pathways and their corresponding gene sets from the KEGG database. Each gene-pathway combination was treated as a distinct feature, resulting in the construction of a cell-by-gene–pathway pair expression matrix. For example, if gene A belongs to pathway I, a column labeled “geneA.PathwayI” was created. Similarly, if both gene A and gene B belong to pathway II, separate features such as “geneA.PathwayII” and “geneB.PathwayII” were generated. Through this approach, we obtained a total of 12,413 unique gene–pathway pair features, where each row represents a single cell and each column represents a specific feature. The matrix values correspond to log₂-transformed TPM values, indicating the expression level of a specific gene within a specific pathway in each cell. Therefore, the numbers presented in the matrix represent the log₂-transformed TPM expression values for the corresponding cells (rows). (DOCX) [file pone.0343633.s001.docx]

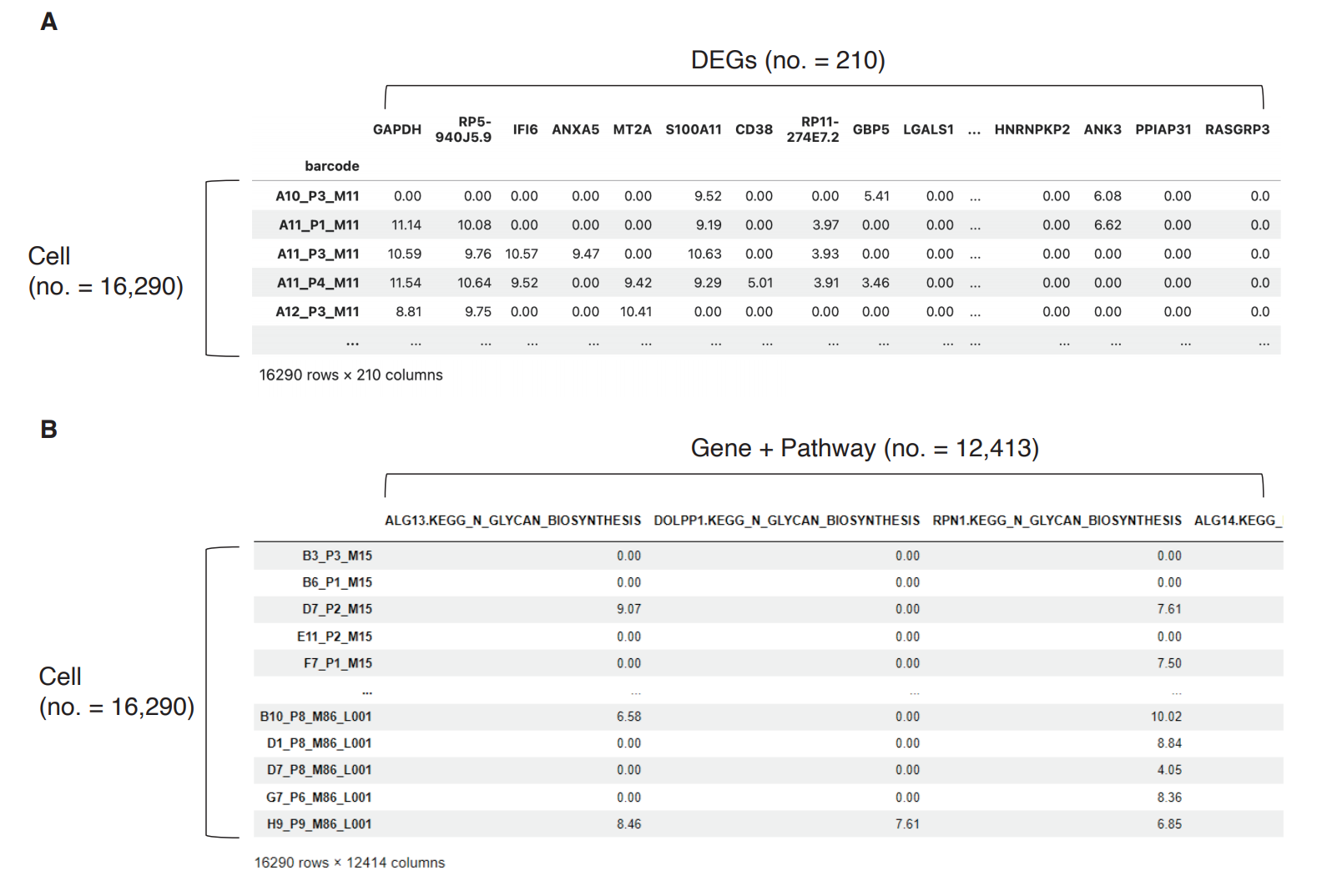


**S1 Fig**. Input data format. (A) Input format of single-cell RNA-seq data for single gene-based modeling using six models. All six models used a same cell × DEG matrix (cell-by-gene matrix) as their input. Specifically, each row represents an individual cell and each column corresponds to selected differentially expressed genes (DEGs), and this gene expression matrix was used as a common input across all models. (B) Input format of single-cell RNA-seq data for pathway-based modeling using 1D-CNN, XGBoost, Random Forest, feed-forward neural network (FNN), logistic regression, and SVM. In this study, we collected 186 pathways and their corresponding gene sets from the KEGG database. Each gene-pathway combination was treated as a distinct feature, resulting in the construction of a cell-by-gene–pathway pair expression matrix. For example, if gene A belongs to pathway I, a column labeled “geneA.PathwayI” was created. Similarly, if both gene A and gene B belong to pathway II, separate features such as “geneA.PathwayII” and “geneB.PathwayII” were generated. Through this approach, we obtained a total of 12,413 unique gene–pathway pair features, where each row represents a single cell and each column represents a specific feature. The matrix values correspond to log₂-transformed TPM values, indicating the expression level of a specific gene within a specific pathway in each cell. Therefore, the numbers presented in the matrix represent the log₂-transformed TPM expression values for the corresponding cells (rows).
