# Supplementary material for: Annotation-free prediction of immunotherapy response in melanoma using single-cell transcriptomic data
Source: PLoS One. 2026 Feb 27;21(2):e0343633. doi: 10.1371/journal.pone.0343633 (PMC12948085; doi:10.1371/journal.pone.0343633)
Supplement: S1 File — S2 Fig. Input format of single-cell RNA sequencing data for pathway-based modeling using the 2D Convolutional Neural Network (2D CNN) model. S3 Fig. Differences in immune cell profiles according to immunotherapy response in single-cell RNA-seq data from malignant melanoma patients treated with immunotherapy. S4 Fig. Expression patterns of top contributing genes from the scRNA-seq based predictive model, stratified by immunotherapy response group (responders vs. non-responders). S5 Fig. Expression patterns of 29 genes across immune cell types derived from scRNA-seq data. S6 Fig. Gene expression pattern of other key genes according to treatment response across three independent melanoma cohorts with bulk RNA-seq data (Wilcoxon rank-sum test). S7 Fig. Feature importance heatmap of responders and non-responders, derived from a 2D Convolutional Neural Network (2D-CNN) model using KEGG pathways. S8 Fig. Expression patterns of genes within the KEGG Chemokine Signaling Pathway (including CCR7) in responders versus non-responders. Even within the same pathway, several genes, such as CCR7, show high expression in responders, whereas others are higher in the non-responders, highlighting intra-pathway heterogeneity. S1 Table. Pathological and clinical characteristics of three distinct melanoma cohorts treated with immunotherapy analyzed in this study. S2 Table. RNA sequencing platforms used in the three distinct melanoma cohorts. S3 Table. Summary statistics of expression for 29 genes in responders and non-responders. S4 Table. Top 10 genes identified by our AI model in relation to immunotherapy responder and non-responders in melanoma. (ZIP) [file pone.0343633.s002.zip › Supplementary figures and tables/S6 Fig.docx]

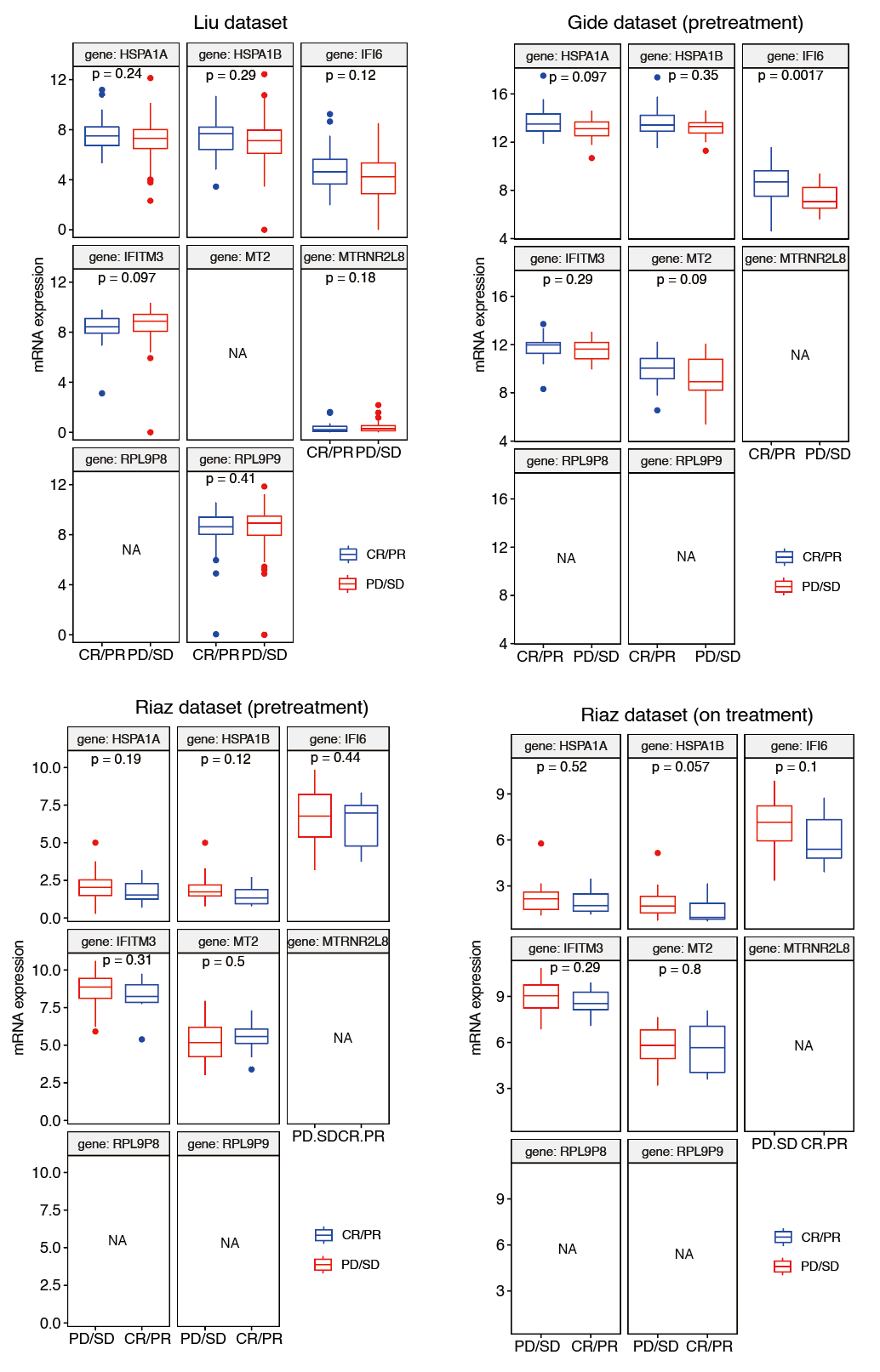


**S6 Fig**. Gene expression pattern of other key genes according to treatment response across three independent melanoma cohorts with bulk RNA-seq data (Wilcoxon rank-sum test).
